# Web-based Self-help Program for Adjustment Problems After an Accident (SelFIT): Protocol for a Randomized Controlled Trial

**DOI:** 10.2196/21200

**Published:** 2020-12-17

**Authors:** Julia Katharina Hegy, Noemi Anja Brog, Thomas Berger, Hansjoerg Znoj

**Affiliations:** 1 Department of Health Psychology and Behavioral Medicine Institute of Psychology University of Bern Bern Switzerland; 2 Department of Clinical Psychology and Psychotherapy Institute of Psychology University of Bern Bern Switzerland

**Keywords:** accidents, adjustment problems, e-mental health, guidance on demand, online, psychological prevention, psychological self-help, study protocol, web-based

## Abstract

**Background:**

Accidents and the resulting injuries are among the world’s biggest health care issues, often causing long-term effects on psychological and physical health. With regard to psychological consequences, accidents can cause a wide range of burdens including adjustment problems. Although adjustment problems are among the most frequent mental health problems, there are few specific interventions available. The newly developed program SelFIT (German acronym: Selber wieder fit nach einem Unfall; “fit again after an accident”) aims to remedy this situation by offering a low-threshold, web-based self-help intervention for psychological distress after an accident.

**Objective:**

The overall aim is to evaluate the efficacy and cost-effectiveness of the SelFIT program plus care as usual (CAU) compared to only CAU. Furthermore, the program’s user-friendliness, acceptance, and adherence are assessed. We expect that the use of SelFIT will be associated with a greater reduction in psychological distress, greater improvement in mental and physical well-being, and greater cost-effectiveness compared to CAU.

**Methods:**

Adults (n=240) experiencing adjustment problems due to an accident they had between 2 weeks and 2 years before entering the study will be randomized into either the intervention or control group. Participants in the intervention group receive direct access to SelFIT. The control group receives access to the program after 12 weeks. There are 6 measurement points for both groups (baseline as well as after 4, 8, 12, 24, and 36 weeks). The main outcome is a reduction in anxiety, depression, and stress symptoms that indicate adjustment problems. Secondary outcomes include well-being, optimism, embitterment, self-esteem, self-efficacy, emotion regulation, pain, costs of health care consumption, and productivity loss, as well as the program’s adherence, acceptance, and user-friendliness.

**Results:**

Recruitment began in December 2019 and will continue at least until January 2021, with the option to extend this for another 6 months until July 2021. As of July 2020, 324 people have shown interest in participating, and 48 people have given their informed consent.

**Conclusions:**

To the best of our knowledge, this is the first study examining a web-based self-help program designed to treat adjustment problems resulting from an accident. If effective, the program could complement the still limited offerings for secondary and tertiary prevention of psychological distress after an accident.

**Trial Registration:**

ClinicalTrials.gov NCT03785912; https://clinicaltrials.gov/ct2/show/NCT03785912

**International Registered Report Identifier (IRRID):**

DERR1-10.2196/21200

## Introduction

### Background

The World Health Organization reports tens of millions of accidents annually, with about 5 million people dying from the consequences of their injuries [1,2]. Accidents and resulting injuries are therefore among the world’s biggest health care issues, often causing long-term effects on psychological and physical health. Due to their unpredictability, uncontrollability, suddenness, and threat to one’s health and integrity, accidents have a high potential for traumatization [3,4]. Medical treatment immediately after the accident and rehabilitation treatments for injured persons have reached a comparatively high standard of care. However, secondary and tertiary prevention of psychological distress are not yet part of routine care [3]. Furthermore, the need for both physical and psychological rehabilitation is growing, and existing services cannot meet the demand [2,5]. Thus, easily available, flexible, and affordable accident rehabilitation and trauma prevention programs are essential to meet the growing demand and improve existing treatment options in accident rehabilitation. Therefore, we have developed SelFIT (German acronym: Selber wieder fit nach einem Unfall; “fit again after an accident”), a low-threshold, web-based psychological self-help program for people who experience psychological distress after an accident. This program will be evaluated in a randomized controlled trial (RCT).

#### Psychological Distress and Adjustment Problems After an Accident

Not everybody who suffers an accident develops psychological problems. Nonetheless, when taking into account possible short-term implications such as fear, pain, or helplessness, as well as potential long-term consequences like permanent physical damage or financial challenges, the development of psychological problems after an accident is easily understandable [6]. Therefore, experiencing an accident can lead to the development of various psychological problems and disorders including anxiety, depression, and post-traumatic stress disorder [6,7]. Stress-related problems such as *adjustment problems* are especially common and frequent among accident victims [8,9]. By adjustment problems, we mean a “maladaptive reaction to a stressful event or ongoing psychosocial difficulties characterized by symptoms of preoccupation with the stressor, recurrent and or distressing thoughts about the stressor, or rumination about its implications” [10]. Adjustment problems can interfere with everyday functioning, cause a loss of interest in different areas of life, and result in an impairment in social or occupational functioning [10]. If persistent, adjustment problems can turn into an adjustment *disorder.* A longitudinal study on adjustment disorder after trauma exposure and major injury conducted in Australia found that participants with adjustment disorder 3 months after the trauma were more likely to meet the criteria for a further psychiatric disorder 12 months post-injury [11]. Thus, the existence of an adjustment disorder heightened the risk for developing other, more serious psychological disorders. Moreover, it was found that the presence of an adjustment disorder increases the risk for suicidality [12]. This highlights the importance of developing and implementing interventions to treat psychological distress after trauma exposure, like experiencing an accident, as early as possible [7,13].

#### Web-based Psychological Interventions

One possibility to implement early interventions for the treatment of psychological distress due to an accident is the development of web-based interventions. Numerous studies have shown that web-based interventions are an effective treatment option for various psychological problems and demographic groups [14,15]. There are many different forms of and applications for such interventions. An important distinguishing factor is guidance, that is, the degree of contact with a health care professional given within the program. Unguided or self-guided programs do not involve any contact with a health care professional, whereas guided programs involve some form of contact or support [16]. Both guided and unguided programs have proven to be effective treatment options. However, the results of several meta-analyses indicate that guided programs tend to yield greater effects than unguided programs [16,17]. This may be explained by a heightened sense of responsibility in the user when in contact with another person compared to nonhuman contact with a machine program [18]. The heightened sense of responsibility can increase adherence, which in turn can be associated with better patient results [19,20]. However, the question arises as to how much and what type of contact is needed to increase adherence and achieve better treatment effects [20].

In this respect, studies on another form of guidance, namely guidance on demand, are of particular interest. With the guidance on demand approach, contact with a professional is only established at a participant’s request but is not scheduled or planned per se.

The findings on the effectiveness of guidance on demand are mixed. In a study on the treatment of tinnitus via the internet, Rheker et al [21] reported that there was no difference between a program version with guidance on demand and an unguided version. Krieger et al [22] used the guidance on demand approach in a web-based intervention for increased self-criticism. Compared to a control group, their results indicate that the treatment with guidance on demand is effective. The guidance on demand approach was also tested by Kleiboer et al [20]. They conducted an RCT on the role of support in a web-based problem-solving treatment for depression and anxiety, comparing 5 different forms and degrees of guidance. Participants either received (1) the program without guidance, (2) the program with guidance on demand, (3) the program with weekly support, (4) no program but nonspecific chat or email support, or (5) allocation to a wait-list control group. Concerning program adherence, the guidance on demand group showed rates comparable to the group with weekly support and significantly higher rates compared to the unguided group. Regarding the treatment effects, however, the guidance on demand group did not show superior effects to the control group [20].

These findings suggest that the guidance on demand approach lies between guided and unguided programs in terms of effectiveness. The approach thus offers a middle way and has the potential to combine some of the most prominent advantages of both guided and unguided treatments: Participants are given the security of knowing that they can turn to a specialist for help and are therefore not completely on their own. However, since no regular contact is scheduled, fewer staff are needed. Thus, programs with on-demand guidance generate lower costs and are less limited to the time and resources of a project’s employees than guided programs. This allows for flexible use at a self-determined pace. Due to the voluntary nature of the contact with a specialist, the participants’ social exposure in a program with guidance on demand can be as low as in an unguided program. Programs with guidance on demand also have other advantages of web-based interventions such as easy availability and scalability (ie, the capacity to increase the number of people who can use the program).

In recent years, various web-based treatment options for adjustment problems and disorders have been developed. One of them is Trastornos Adaptivos Online [23,24]. The guided program comprises psychoeducational elements, strategies from positive psychology, and techniques to manage negative emotions and improve problem solving. In addition to the program, participants receive short weekly therapist support via telephone. Trastornos Adaptivos Online was well received by both clinicians and patients in a pilot study [24]. The program is currently being tested for its effectiveness in an RCT [23].

A further web-based intervention for the treatment of adjustment disorders is the Brief Adjustment Disorder Intervention [25,26]. This program is unguided and consists of four modules, which the participants can process in a self-determined order. The program’s theoretical approach is mainly CBT-based but also contains elements of mindfulness as well as findings from research on stress and coping [27]. Preliminary results of an RCT indicate that participants who used the program at least once within a month showed a decrease in symptoms of adjustment disorders and an increase in psychological well-being. However, there was a very high dropout rate, which is mentioned as the study’s most prominent limitation [26]. The authors tested the effects of additional therapist support on the program’s effectiveness. The additional support did not contribute significantly to the study’s outcomes [26]. This supports previous findings by Maercker et al [28] that web-based self-help interventions may be a promising treatment option for adjustment problems and further indicates that such interventions do not necessarily need scheduled guidance from a specialist.

Another unguided web-based program for adjustment problems is ZIEL (German acronym: Zurück Ins Eigene Leben; “back to your own life”) [29]. The ZIEL program comprises different evidence-based techniques from treatments for depression, anxiety disorders, and post-traumatic stress disorders. ZIEL consists of 5 sections which participants can work through freely and as needed over a course of 4 weeks. In an RCT, the participants of both the experimental and the control group showed an improvement in the severity of symptoms of adjustment problems. The intervention group, however, showed a significantly greater improvement in terms of depressive symptoms and quality of life. However, the authors of ZIEL also report challenges with a high dropout rate and suggest different measures to address this. One of these suggestions is to focus on certain subgroups of people with adjustment problems or on certain triggers of adjustment problems. This, in turn, would allow for a more tailored response to the needs of the users, thereby creating a better user-program fit and greater relevance for the users [29].

#### Rationale

Against the background that accidents can have various long-term psychological consequences such as adjustment problems, which are often not or insufficiently treated, we have developed SelFIT. The program was realized as a web-based program in order to provide an easily accessible psychological treatment option to accident victims. Considering the results and conclusions from previous research on web-based self-help interventions for adjustment problems described above, SelFIT was not created as a treatment for adjustment problems in general, but specifically for the treatment of psychological distress and adjustment problems after an accident. This focus allows for more specific thematic tailoring to the needs of the target population. Additionally, the guidance on demand approach was chosen in order to take advantage of as many benefits of guided and unguided programs as possible without generating excessive additional costs.

### Aims and Objectives

The aim of this study is to conduct a randomized controlled trial to evaluate the new SelFIT program developed for people who experience adjustment problems after an accident. Specifically, the objectives are to evaluate the efficacy and cost-effectiveness of SelFIT used in addition to care as usual (CAU) compared to only CAU; to analyze the acceptance and user-friendliness of the SelFIT program and draw conclusions for further development of the program and the type of guidance applied in the program (ie, guidance on demand); and to explore and analyze moderators (eg, age, sex, or satisfaction with the program), mediators (eg, adherence), and predictors (eg, adherence, embitterment, or optimism) for the efficacy of the program.

## Methods

### Study Design

#### Overview

This study is a prospective longitudinal RCT. The study population are German-speaking adults (≥18 years) who suffer from adjustment problems after experiencing an accident between 2 weeks and 2 years before entering the study.

The lower time limit was set in order to reach injured persons as soon as possible after the accident and thereby prevent the development or worsening of psychological distress such as adjustment problems. The upper time limit was set in accordance with the time criterion of a chronic adjustment disorder according to the current version of the International Classification of Diseases (ICD-10 [30]).

**Figure 1:**
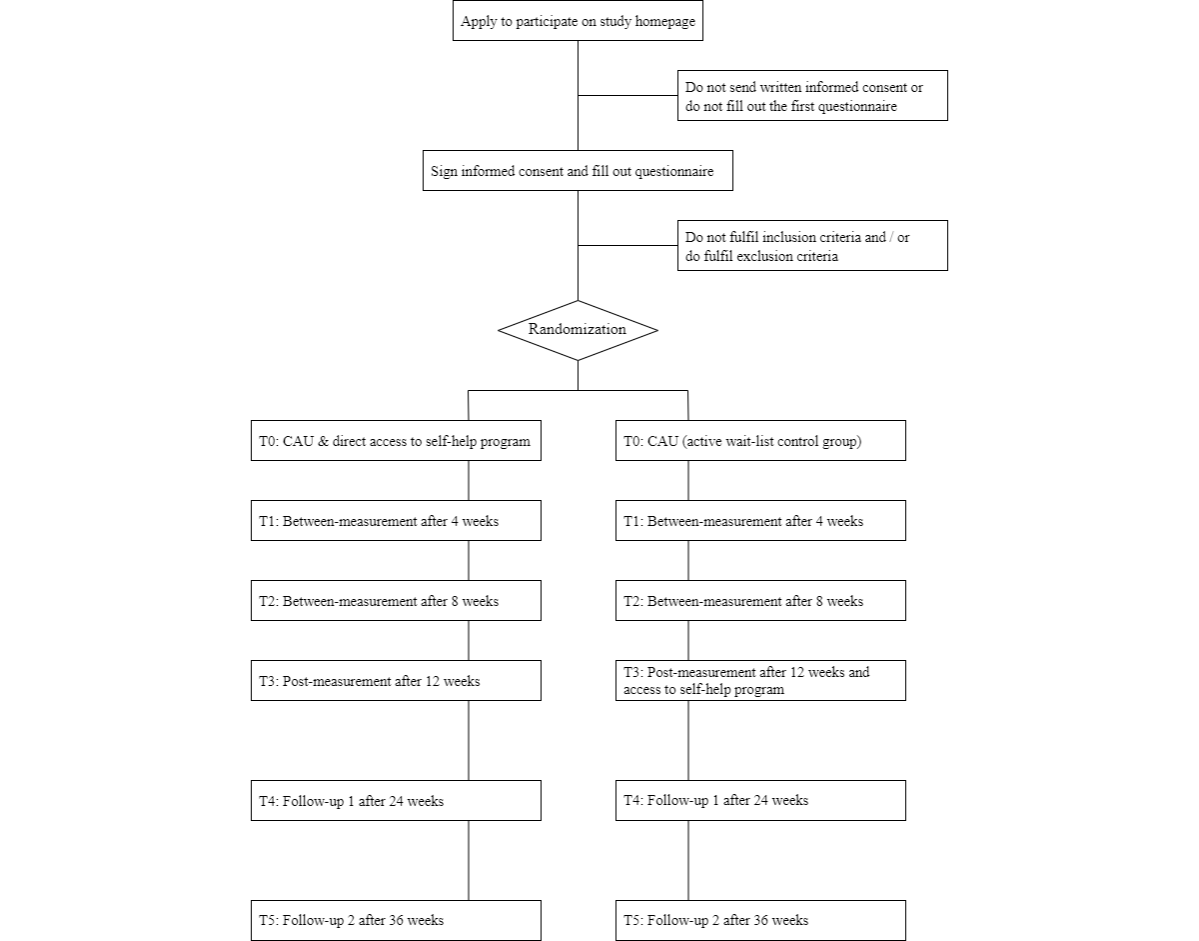
Flowchart of the SelFIT study design. CAU: care as usual.

Figure 1 displays the study flowchart, illustrating that participants in the intervention group receive direct access, while those in the control group receive access after 12 weeks. All participants are asked to complete the online assessment at 6 time points. The first questionnaire (premeasurement) serves both as a baseline and as the screening for inclusion and exclusion criteria. There are two between-measurements after 4 and 8 weeks. The postmeasurement takes place after 12 weeks. All participants are asked to participate in the follow-up measurements taking place 24 and 36 weeks after randomization to evaluate the long-term effects of the intervention. Participants who drop out at any point will be asked to participate in any remaining measurements nonetheless.

#### Randomization

After receipt of the informed consent and the initial screening, participants are randomized equally (1:1 ratio) into the treatment or the control group. Randomization is stratified by the point value that participants score on the questionnaire used as the primary outcome measure (12-16 points vs ≥17 points) in order to make the two groups comparable regarding their symptom expression on this measure. This is done with a computerized random number generator and randomly permuted block sizes using Randomization.com [31]. Randomization within each stratum uses a 1:1 ratio as well. The allocation schedule is generated by a researcher not involved in the research process and is unknown to the investigators.

### Recruitment

#### Overview

Recruitment takes place via advertising on social media, websites, and internet forums, and with different organizations and self-help groups, as well as through referrals from rehabilitation clinics, psychotherapeutic practices and clinics, physiotherapists, medical doctors, and hospitals. People interested in participating can leave their contact details on the study home page and will then be sent the participant information either by email or by post, according to their choice. After the signed informed consent form is sent to the study team, potential participants are asked to complete the first online questionnaire to check that they do not meet any of the exclusion criteria and meet all the inclusion criteria described below.

#### Eligibility Criteria

According to the inclusion criteria of this study, all participants must have experienced and be able to specify an accident during the period from 2 weeks to 2 years prior to participation in this study; exceed the cutoff value for at least a mild psychological burden on the 21-item Depression, Anxiety and Stress Scale [32]; be at least 18 years old; provide informed consent; have access to the Internet; have mastered the German language; and be able to specify an emergency address in the event of an acute crisis.

Persons who show severe depressive symptoms (Beck Depression Inventory II score>29) [33], show suicidal tendencies (Beck Depression Inventory II suicide item>1), or have a known diagnosis of a psychotic or bipolar disorder are excluded.

### Description of the Intervention

The SelFIT program takes 12 weeks in total. It consists of an introduction, 8 thematic modules, and a conclusion. The thematic modules are described in Textbox 1. Furthermore, the program includes a page with information about the procedure to be followed in emergencies and acute crises as well as a list of suitable contacts in such situations. Multimedia Appendix 1 shows a screenshot of the program’s home page.

Participants are encouraged to work on one module per week and to repeat and deepen the various exercises during the last 4 weeks of the program in order to facilitate the transfer to their own everyday life. However, participants are free to choose both the order and speed of processing of the modules themselves. All modules consist of a video, various texts, exercises, and weekly tasks. In addition, participants are asked to indicate which of a list of feelings, moods, and physical conditions they currently experience. This allows them to observe how their well-being changes over the course of the program.

Since the study employs a guidance on demand approach, participants can contact the study team if needed or desired. For this purpose, they can either write an email or use the chat function within the program. In the settings, there is also the option to choose whether participants want to receive a reminder email after a certain period of inactivity. Other than this, contact with the study team, a therapist, or a counsellor is not planned by default.

**Outline of the thematic modules of the SelFIT program.**

**Module 1: Accidents and their consequences**

- Information about psychological and physical consequences of accidents as well as the symptoms of adjustment problems
- Survey of the participant's current situation and well-being

**Module 2: Changing perspectives**

- Information on automatic and irrational assumptions, chains of thoughts, and the influence of thoughts and assumptions on one's state of mind
- Exercises with the aim of cognitive restructuring

**Module 3: Understanding different reactions to accidents**

- Information about frequent psychological reactions to accidents
- Exercise to identify physical symptoms of anxiety

**Module 4: Activation**

- Behavioral activation with suggestions for different types of activation
- Development of a personal activity plan
- Information about the importance of physical activity

**Module 5: Self-care**

- Information about post-traumatic growth
- Exercises to promote acceptance, gratitude for positive aspects of life and personal resources

**Module 6: Finding calm**

- Information about sleep and sleep hygiene
- Exercises to promote mental and physical relaxation

**Module 7: Addressing painful feelings**

- Information about typical reasoning errors
- Information about and exercises for dealing with painful feelings such as guilt, shame, anger, and resentment after an accident

**Module 8: Self-efficacy**

- Information about attribution styles, self-fulfilling prophecies and self-instructions
- Identification and activation of personal resources
- Exercise to promote self-confidence

### Measures

**Figure 2:**
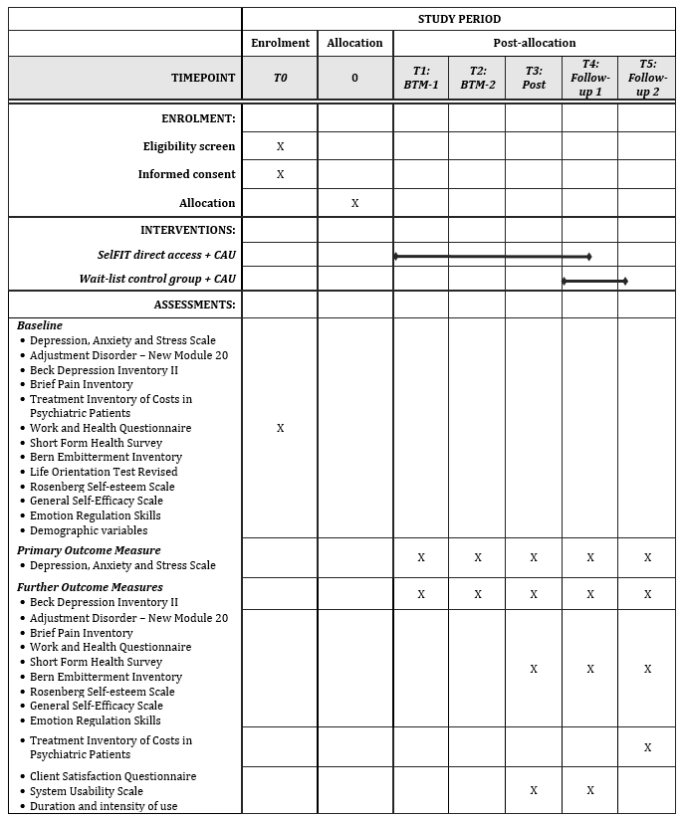
SPIRIT (Standard Protocol Items: Recommendations for Interventional Trials) figure to display the study’s schedule of enrolment, interventions, and assessments. BTM: between-measurement. CAU: care as usual.

All instruments used over the course of the study are self-report questionnaires that are completed online. Figure 2 gives an overview of all questionnaires with the time points of the assessments. Since the study population is German-speaking, we use the German version for all questionnaires.

#### Primary Outcome Measure

The primary outcome measure is the short version of the Depression, Anxiety and Stress Scale with 21 items [32]. Each of this questionnaire’s three scales contains 7 items assessing symptoms of depression, anxiety and stress on a 4-point Likert scale ranging from 0=never to 3=almost always. The 21-item Depression, Anxiety and Stress Scale is not diagnosis-specific and has proven to be a well-suited measure of psychological distress in a broad range of clinical and nonclinical samples [34,35]. This is why we use this questionnaire to assess symptoms of psychological distress and adjustment problems.

### Further Outcome Measures

#### Adjustment Problems and Depressive Symptoms

Adjustment problems are also assessed using the Adjustment Disorder–New Module 20 [36]. The questionnaire was designed according to the upcoming ICD-11 symptom definition of adjustment disorder. It consists of 20 items, which are divided into a stressor list and an item list. While the stressor list captures different acute and chronic life events, the item list assesses the symptoms occurring in response to those stressors. In accordance with the ICD-11 definition of adjustment disorder these symptoms are based on the adjustment disorder core symptoms of avoidance, anxiety, impulse disturbance and depressive mood [36].

Depressive symptoms are additionally assessed using the Beck Depression Inventory II [33]. This questionnaire consists of 21 items, which are rated on a Likert scale ranging from 0=not at all to 3=very strong. Item 9 of this instrument is also used to screen for suicidality at all 6 measurement points of the study.

#### Accident-Related Measures

Regarding the accident, we ask for a short description of the event, the time that has passed since, and its subjectively perceived severity.

Pain due to the accident is assessed by the Brief Pain Inventory [37], which consists of 15 items. After the question of whether there is any pain that exceeds the normal levels expected in everyday life, participants have to indicate where the pain is located and how severe the limitations in various areas of life are due to the pain. We also assess the participants’ own perception of their physical attractiveness. Physical attractiveness is a very influential informational cue that is used frequently and consistently [38,39]. It has been shown to play an important role in how a person is perceived and responded to in many areas of life [38]. This includes health and psychological well-being. Bordieri et al [40] found that physical attractiveness influenced how others make attributions concerning the cause and prognosis of someone’s disability. Physical attractiveness was also linked to self-esteem [40]. Among the possible consequences of an accident is skin scarring. In most cases, this is perceived as unattractive [41]. Brown et al [41] found that skin scars impact a person’s acceptability to others as well as themselves and also have an effect on social functioning and emotional well-being. Thus, participants in this study are asked to rate their own physical attractiveness compared to that of other people their age. They also have to indicate how often they think about being rated by others in terms of physical attractiveness. Furthermore, the participants assess if and how much their own perception of their physical attractiveness has changed since the accident.

#### Cost-effectiveness Measures

Two questionnaires, each with a different emphasis, are used to assess the cost-effectiveness of the SelFIT program. The costs of health care consumption and productivity loss are assessed using the Treatment Inventory of Costs in Psychiatric Patients [42], a self-report questionnaire with 23 items of varying answer formats. Work-related factors with regard to the consequences of the accident are assessed by means of the Work and Health Questionnaire [43]. This questionnaire consists of 3 different parts with a total of 21 items with varying answer formats. The first part contains 5 items on current work activity, the second part contains 7 items on workload and cooperation, and the third part contains 9 items on health and well-being.

#### Embitterment and Optimism

We also assess embitterment and optimism. The Bern Embitterment Inventory [44], an 18-item questionnaire with answers ranging from 0=not at all true to 4=exactly true, is used to survey embitterment. Optimism is assessed as a predictor at the baseline measurement with the Life Orientation Test Revised [45], which consists of 10 items with answer categories ranging from 0=strongly disagree to 4=strongly agree.

General well-being and the ability to cope in everyday life are assessed by the Short Form 12 Health Survey [46]. The questionnaire consists of 12 items with different answer designs and options.

#### Self-esteem, Self-efficacy, and Emotion Regulation Skills

Furthermore, we assess self-esteem, self-efficacy and emotion regulation skills. Self-esteem is measured using the Rosenberg Self-esteem Scale [47]. This scale consists of 10 items with a 4-point scale from 0=strongly agree to 3=strongly disagree.

The General Self-Efficacy Scale [48] serves as a measure to assess perceived self-efficacy, aiming to predict coping with daily hassles and general adjustment after experiencing a stressful life event. The scale comprises 10 items on a scale from 1=not at all true to 4=exactly true. Emotion regulation skills are assessed via the Self-Report Measure for the Assessment of Emotion Regulation Skills [49], a 27-item questionnaire with answers ranging from 0=not at all to 3=(almost) always.

#### Program-Related Measures

The program-related factors we survey include user satisfaction and program usability. This is assessed using the Client Satisfaction Questionnaire [50,51], an 8-item scale with a 4-tiered answer format varying in its wording. The program’s usability is measured with the System Usability Scale [52], which consists of 10 items with answers ranging from 1=do not agree at all to 5=completely agree.

A further program-related factor is adherence, measured by the frequency and duration of use. Those parameters are gathered within the program by means of, for example, the number of log-ins or the percentage of pages and segments that have been accessed and browsed through at least once.

#### Demographic Variables

Demographic variables obtained from the first online questionnaire include gender, age, family status, and level of income and education. Additionally, participants are asked to indicate whether they have received or currently receive any treatment for mental health issues or physical rehabilitation.

### Data Collection and Management

All data is assessed online, either within the program platform or via online questionnaires programmed in Qualtrics [53]. Data integrity is enforced through different mechanisms including referential data rules, valid values, range checks, and consistency checks. The option to choose a value from a list of valid codes and a description of what each code means will be available where applicable. Checks are applied at the time of data entry into a specific field. All data is stored in anonymous form and can only be traced by a code that cannot be linked to the identity of the participant. Data gathered within the program as well as the program itself are stored on a firewall-encrypted backed-up server of the University of Bern. Only researchers directly involved in the study have access to the data, and they are subject to professional discretion.

### Power

In order to specify the sample size needed for the planned analyses, we conducted a power analysis based on a probability level of .05 and a power of 0.80 using G*Power (Heinrich-Heine-Universität Düsseldorf) [54]. To test the program’s efficacy compared to the control group, we expect small-to-moderate effect sizes between *d*=0.2 and *d*=0.35 as well as a correlation between the groups of *r*=0.4. Those estimates are based on the results of previous web-based interventions for adjustment problems [29].

The a priori power analyses yielded a necessary sample size of 80 (for *d*=0.35) to 238 (for *d*=0.2) participants in total for this analysis. Since the program does not include weekly guidance but rather guidance on demand, we expect slightly smaller effect sizes. Based on these calculations and assumptions we decided to target a sample size of N=240 participants.

### Statistical Analysis

Statistical analyses will be carried out on the basis of the intention-to-treat approach and therefore will include all randomized participants. We will analyze the extent of the missing data, explore patterns and determine the type of missing data (missing completely at random, missing at random, not missing at random). Missing values will be substituted using multiple imputations. Sensitivity analyses will be conducted for both the data sets with and without the imputed data.

We will use linear mixed models to analyze all continuous outcomes as a change from baseline to compare effects between the two groups and over the different measurement points. In case of a missing at random mechanism, we will conduct multilevel regression analyses, which are less sensitive to missing data. Multiple regression analyses allow us to include several predictors such as time of measurement or group allocation [55].

Furthermore, exploratory analyses will be conducted. One of these will examine the association between adherence and outcome, since a higher adherence has been shown to have a positive effect on outcome [56,57].

All analyses will be conducted using SPSS (IBM Corporation) and R (R Foundation for Statistical Computing).

## Results

The study is conducted according to the principles of the World Medical Assembly Declaration of Helsinki [58], the Swiss Federal Human Research Act [59], and the Ordinance on Clinical Trials in Human Research [60]. Ethical approval has been obtained from the Cantonal Ethics Committee Berne (BASEC 2018-01059). The study is registered with ClinicalTrials.gov (NCT03785912). Furthermore, the SelFIT program is a CE-certified medical device. Written informed consent is obtained from each participant.

Recruitment, screening, inclusion, and randomization of participants is scheduled to take place between December 2019 and January 2021. Due to the COVID-19 pandemic, recruitment was slowed down. For this reason, the recruitment phase may be extended by 6 months if we are unable to recruit enough participants by the end of January 2021.

**Figure 3:**
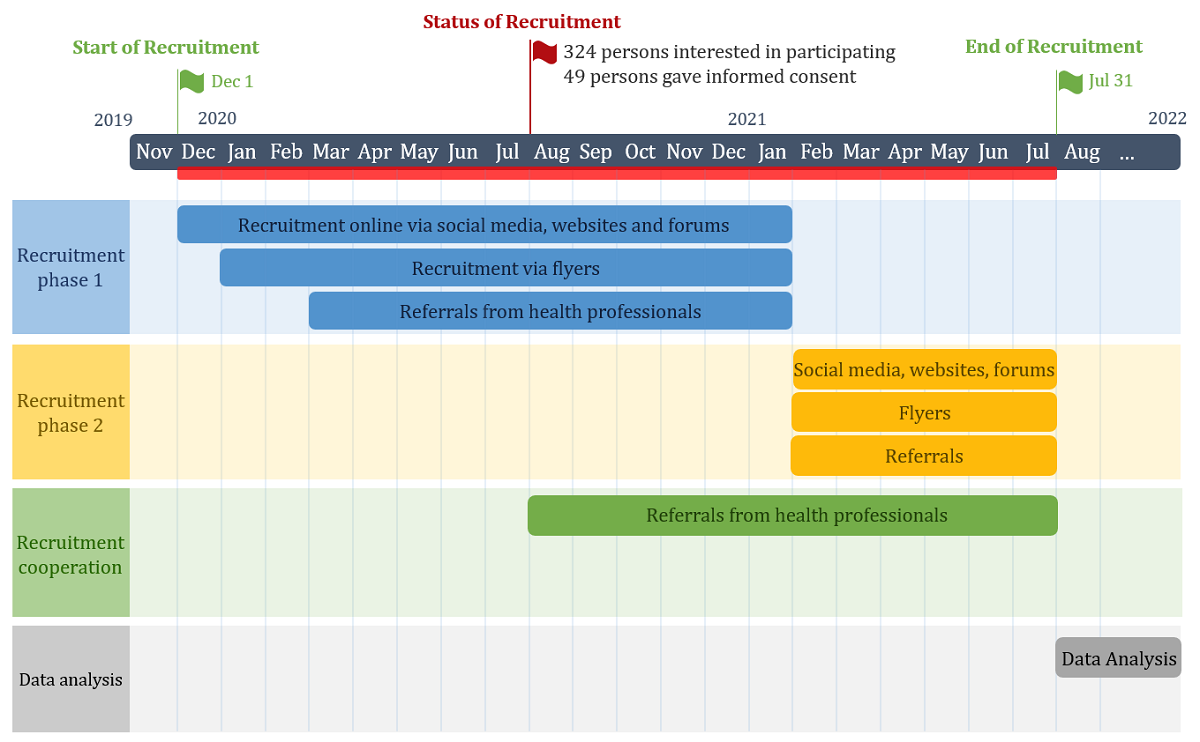
Gantt chart displaying the study’s recruitment schedule and status of recruitment.

As of July 2020, 324 people have shown interest in participating, and 49 people have given their informed consent. We are confident that we will be able to recruit enough people for three reasons. First, a recruitment cooperation with one of the largest rehabilitation clinics for injured persons in Switzerland will start in August. This means a steady influx of participants. Second, all persons who have expressed interest in participating but have not registered are contacted again and asked if they would like to participate in the study. This has proven to be an effective strategy so far. Third, we can extend the recruitment phase if necessary. The project’s recruitment schedule is shown in Figure 3.

## Discussion

### Principal Findings

Accidents and their consequences often affect not only a person's physical well-being but also their mental health and their professional, personal, and social environment. Thus, accidents often mean a significant change and new challenges for those affected. Adjusting to those changed circumstances can be difficult. This also applies to adjustment efforts when returning to everyday life after rehabilitation is over, for example. A lack of support during this time may lead to the development or worsening of psychological distress such as adjustment problems. Easily accessible treatment options such as the SelFIT program could remedy this situation. For this reason, SelFIT was implemented as a web-based self-help intervention, which allows a high degree of flexibility in terms of time and location and can be used with comparatively little effort. Based on the findings of previous studies on adjustment problems and web-based interventions, SelFIT comprises a guidance on demand approach. This enables users to obtain support when needed with minimal personnel costs. Unlike previous web-based programs on adjustment problems, SelFIT does not address adjustment problems in general. Instead, the focus is specifically on the treatment of psychological distress and adjustment problems after an accident. This allows the content of the program to be matched more specifically to the needs of the participants.

To the best of our knowledge, this combination of web-based delivery, guidance on demand, and focus on the psychological support of injured persons has not yet been done.

Due to this novel approach, SelFIT could also contribute to expanding the scope of therapy options and offers of clinics, practices, or hospitals. Here, the program could serve as a supplement to face-to-face therapy. In addition, SelFIT can also offer extended psychological support, for example after the end of a rehabilitation program in the transition to everyday life at home or as a transitional offer after the end of psychotherapy.

The results of this study will provide insight into the efficacy and cost-effectiveness of the SelFIT program. The analysis of the program’s user-friendliness and adherence may provide information for further adaptations to different user needs.

### Limitations

Possible limitations of this study include typical challenges of web-based interventions such as the self-selection of participants. This is addressed by recruiting through various channels, such as program referrals from a rehabilitation clinic for injured people, recruitment via social media, and recommendations by physiotherapists. Another potential limitation is participants’ physical restrictions due to the accident, which may make it difficult to use a computer or other technical devices. In such a case, access to the program might be limited.

The narrow focus of participants to be included in the study can have disadvantages. Although this allows for more specific tailoring, it makes the target population significantly smaller and recruitment more difficult.

## Appendix

Multimedia Appendix 1 Screenshot of the SelFIT program's homepage.

## Abbreviations

CAU: care as usual
ICD: International Classification of Diseases
RCT: randomized controlled trial
SelFIT: Selber wieder fit nach einem Unfall (fit again after an accident)
ZIEL: Zurück Ins Eigene Leben (back to your own life)

## References

[1] Arienti C, Gimigliano F, Pollet J, Kiekens C, Negrini S (2018). [Cochrane and World Health Organization "Rehabilitation 2030: a call for action"]. Recenti Prog Med.

[2] Gimigliano F, Negrini S (2017). The World Health Organization "Rehabilitation 2030: a call for action". Eur J Phys Rehabil Med.

[3] Angenendt J (2014). [Interventions for mental health sequelae of accidents]. Bundesgesundheitsblatt Gesundheitsforschung Gesundheitsschutz.

[4] Angenendt J, Nyberg E, Frommberger U, Maercker A (2013). Posttraumatische Belastungsreaktionen bei Verkehrsunfallopfern. Posttraumatische Belastungsstörungen.

[5] Angenendt J, Drechsel-Schlund C, Südkamp N, Berger M (2016). Notfallmedizin und Unfallchirurgie: Psychotraumatologie nach Unfällen. Dtsch Arztebl International.

[6] Kühn M (2005). Die psychischen Folgen von schweren Unfällen und ihre Prädiktoren.

[7] Giummarra MJ, Lennox A, Dali G, Costa B, Gabbe BJ (2018). Early psychological interventions for posttraumatic stress, depression and anxiety after traumatic injury: A systematic review and meta-analysis. Clin Psychol Rev.

[8] Shih RA, Schell TL, Hambarsoomian K, Belzberg H, Marshall GN (2010). Prevalence of posttraumatic stress disorder and major depression after trauma center hospitalization. J Trauma.

[9] Bryant RA, Nickerson A, Creamer M, O'Donnell M, Forbes D, Galatzer-Levy I, McFarlane AC, Silove D (2015). Trajectory of post-traumatic stress following traumatic injury: 6-year follow-up. Br J Psychiatry.

[10] Ben-Ezra M, Mahat-Shamir M, Lorenz L, Lavenda O, Maercker A (2018). Screening of adjustment disorder: Scale based on the ICD-11 and the Adjustment Disorder New Module. J Psychiatr Res.

[11] O'Donnell ML, Alkemade N, Creamer M, McFarlane AC, Silove D, Bryant RA, Felmingham K, Steel Z, Forbes D (2016). A Longitudinal Study of Adjustment Disorder After Trauma Exposure. Am J Psychiatry.

[12] Casey P, Jabbar F, O'Leary E, Doherty AM (2015). Suicidal behaviours in adjustment disorder and depressive episode. J Affect Disord.

[13] Forneris CA, Gartlehner G, Brownley KA, Gaynes BN, Sonis J, Coker-Schwimmer E, Jonas DE, Greenblatt A, Wilkins TM, Woodell CL, Lohr KN (2013). Interventions to prevent post-traumatic stress disorder: a systematic review. Am J Prev Med.

[14] Andersson G, Titov N (2014). Advantages and limitations of Internet-based interventions for common mental disorders. World Psychiatry.

[15] Cuijpers P, Donker T, Johansson R, Mohr DC, van SA, Andersson G (2011). Self-guided psychological treatment for depressive symptoms: a meta-analysis. PLoS One.

[16] Baumeister H, Reichler L, Munzinger M, Lin J (2014). The impact of guidance on Internet-based mental health interventions — A systematic review. Internet Interventions.

[17] Andersson G, Cuijpers P (2009). Internet-based and other computerized psychological treatments for adult depression: a meta-analysis. Cogn Behav Ther.

[18] Mohr DC, Duffecy J, Ho J, Kwasny M, Cai X, Burns MN, Begale M (2013). A randomized controlled trial evaluating a manualized TeleCoaching protocol for improving adherence to a web-based intervention for the treatment of depression. PLoS One.

[19] Donkin L, Christensen H, Naismith SL, Neal B, Hickie IB, Glozier N (2011). A systematic review of the impact of adherence on the effectiveness of e-therapies. J Med Internet Res.

[20] Kleiboer A, Donker T, Seekles W, van SA, Riper H, Cuijpers P (2015). A randomized controlled trial on the role of support in Internet-based problem solving therapy for depression and anxiety. Behav Res Ther.

[21] Rheker J, Andersson G, Weise C (2015). The role of “on demand” therapist guidance vs. no support in the treatment of tinnitus via the internet: A randomized controlled trial. Internet Interventions.

[22] Krieger T, Reber F, von Glutz B, Urech A, Moser CT, Schulz A, Berger T (2019). An Internet-Based Compassion-Focused Intervention for Increased Self-Criticism: A Randomized Controlled Trial. Behav Ther.

[23] Rachyla I, Pérez-Ara Marian, Molés Mar, Campos D, Mira A, Botella C, Quero S (2018). An internet-based intervention for adjustment disorder (TAO): study protocol for a randomized controlled trial. BMC Psychiatry.

[24] Rachyla I, Quero S, Pérez-Ara M, Molés M, Campos D, Mira A (2017). Web-based, self-help intervention for adjustment disorders: Acceptance and Usability. Annual Review of CyberTherapy and Telemedicine.

[25] Skruibis P, Eimontas J, Dovydaitiene M, Mazulyte E, Zelviene P, Kazlauskas E (2016). Internet-based modular program BADI for adjustment disorder: protocol of a randomized controlled trial. BMC Psychiatry.

[26] Eimontas J, Rimsaite Z, Gegieckaite G, Zelviene P, Kazlauskas E (2018). Internet-Based Self-Help Intervention for ICD-11 Adjustment Disorder: Preliminary Findings. Psychiatr Q.

[27] Skruibis P, Eimontas J, Dovydaitiene M, Mazulyte E, Zelviene P, Kazlauskas E (2016). Internet-based modular program BADI for adjustment disorder: protocol of a randomized controlled trial. BMC Psychiatry.

[28] Maercker A, Bachem RC, Lorenz L, Moser CT, Berger T (2015). Adjustment Disorders Are Uniquely Suited for eHealth Interventions: Concept and Case Study. JMIR Ment Health.

[29] Moser C, Bachem R, Berger T, Maercker A (2019). ZIEL: Internet-Based Self-Help for Adjustment Problems: Results of a Randomized Controlled Trial. J Clin Med.

[30] World Health Organization (1992). The ICD-10 Classification of Mental and Behavioural Disorders: clinical descriptions and diagnostic guidelines.

[31] Dallal GE (2008). Randomization.com.

[32] Lovibond S, Lovibond P (1995). Manual for the Depression Anxiety Stress Scales (DASS).

[33] Beck A, Steer R, Ball R, Ranieri W (1996). Comparison of Beck Depression Inventories -IA and -II in psychiatric outpatients. J Pers Assess.

[34] Osman A, Wong JL, Bagge CL, Freedenthal S, Gutierrez PM, Lozano G (2012). The Depression Anxiety Stress Scales-21 (DASS-21): further examination of dimensions, scale reliability, and correlates. J Clin Psychol.

[35] Szabó M (2010). The short version of the Depression Anxiety Stress Scales (DASS-21): factor structure in a young adolescent sample. J Adolesc.

[36] Lorenz L, Bachem RC, Maercker A (2016). The Adjustment Disorder–New Module 20 as a Screening Instrument: Cluster Analysis and Cut-off Values. Int J Occup Environ Med.

[37] Cleeland CS, Ryan KM (1994). Pain assessment: global use of the Brief Pain Inventory. Ann Acad Med Singapore.

[38] Patzer GL (1985). The Physical Attractiveness Phenomena.

[39] Patzer GL (1995). Self-esteem and physical attractiveness. J Esthet Dent.

[40] Bordieri JE, Sotolongo M, Wilson M (1983). Physical attractiveness and attributions for disability. Rehabilitation Psychology.

[41] Brown BC, McKenna SP, Siddhi K, McGrouther DA, Bayat A (2008). The hidden cost of skin scars: quality of life after skin scarring. J Plast Reconstr Aesthet Surg.

[42] Bouwmans C, De Jong K, Timman R, Zijlstra-Vlasveld M, Van der Feltz-Cornelis C, Tan SS, Hakkaart-van RL (2013). Feasibility, reliability and validity of a questionnaire on healthcare consumption and productivity loss in patients with a psychiatric disorder (TiC-P). BMC Health Serv Res.

[43] Abegglen S, Hoffmann-Richter U, Schade V, Znoj H (2017). Work and Health Questionnaire (WHQ): A Screening Tool for Identifying Injured Workers at Risk for a Complicated Rehabilitation. J Occup Rehabil.

[44] Znoj H (2008). Berner Verbitterungs-Inventar. Manual.

[45] Herzberg PY, Glaesmer H, Hoyer J (2006). Separating optimism and pessimism: a robust psychometric analysis of the revised Life Orientation Test (LOT-R). Psychol Assess.

[46] Ware J, Kosinski M, Keller SD (1996). A 12-Item Short-Form Health Survey: construction of scales and preliminary tests of reliability and validity. Med Care.

[47] von Collani G, Herzberg PY (2003). Eine revidierte Fassung der deutschsprachigen Skala zum Selbstwertgefühl von Rosenberg. Zeitschrift für differentielle und diagnostische Psychologie.

[48] Jerusalem M, Schwarzer R, Leibniz-Zentrum für Psychologische Information und Dokumentation (ZPID) (2003). SWE. Skala zur allgemeinen Selbstwirksamkeitserwartung [Verfahrensdokumentation aus PSYNDEX Tests-Nr. 9001003, Autorenbeschreibung und Fragebogen]. Elektronisches Testarchiv.

[49] Berking M, Znoj H (2008). Entwicklung und Validierung eines Fragebogens zur standardisierten Selbsteinschätzung emotionaler Kompetenzen (SEK-27). Zeitschrift für Psychiatrie, Psychologie und Psychotherapie.

[50] Attkisson CC, Zwick R (1982). The client satisfaction questionnaire. Psychometric properties and correlations with service utilization and psychotherapy outcome. Eval Program Plann.

[51] Schmidt J, Lamprecht F, Wittmann WW (1989). [Satisfaction with inpatient management. Development of a questionnaire and initial validity studies]. Psychother Psychosom Med Psychol.

[52] Brooke J, Jordan PW, Thomas B, McClelland IL, Weerdmeester B (1996). System Usability Scale (SUS): A Quick-and-Dirty Method of System Evaluation User Information. Usability Evaluation in Industry.

[53] Qualtrics Qualtrics. May 2020 ed.

[54] Faul F, Erdfelder E, Lang AG, Buchner A (2007). G*Power 3: a flexible statistical power analysis program for the social, behavioral, and biomedical sciences. Behav Res Methods.

[55] Brodbeck J, Berger T, Znoj H (2017). An internet-based self-help intervention for older adults after marital bereavement, separation or divorce: study protocol for a randomized controlled trial. Trials.

[56] Fuhr K, Schröder J, Berger T, Moritz S, Meyer B, Lutz W, Hohagen F, Hautzinger M, Klein JP (2018). The association between adherence and outcome in an Internet intervention for depression. J Affect Disord.

[57] Simpson HB, Maher MJ, Wang Y, Bao Y, Foa EB, Franklin M (2011). Patient adherence predicts outcome from cognitive behavioral therapy in obsessive-compulsive disorder. J Consult Clin Psychol.

[58] World Medical Association (2013). World Medical Association Declaration of Helsinki: ethical principles for medical research involving human subjects. JAMA.

[59] (2014). Bundesgesetz über die Forschung am Menschen, SR 810.30.

[60] (2014). Verordnung über klinische Versuche in der Humanforschung, SR 810.305 KlinV.

