# Supplementary figures and images for: Web-based Self-help Program for Adjustment Problems After an Accident (SelFIT): Protocol for a Randomized Controlled Trial
Source: JMIR Res Protoc. 2020 Dec 17;9(12):e21200. doi: 10.2196/21200 (PMC7775196; doi:10.2196/21200)

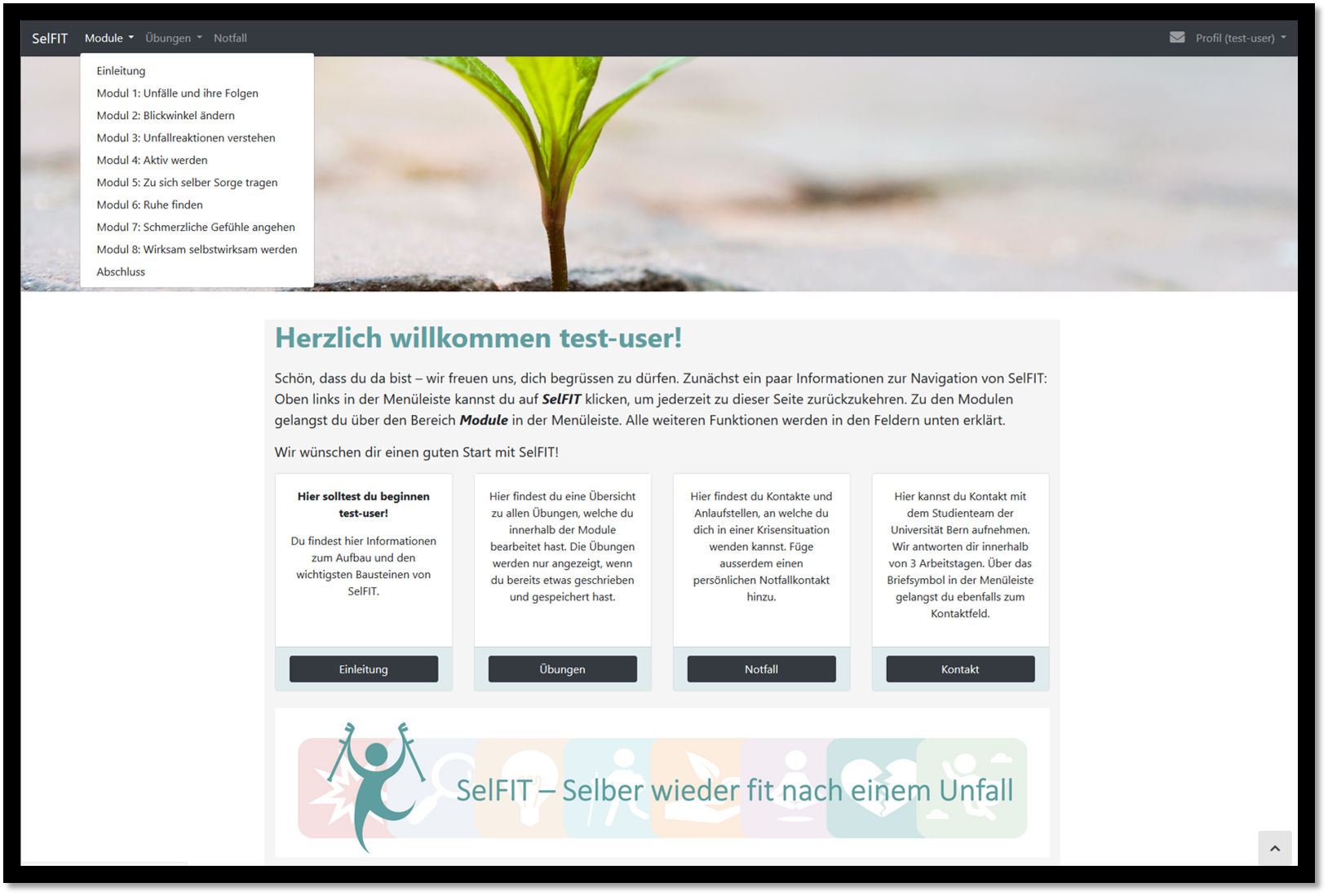

Supplement: Multimedia Appendix 1 [file resprot_v9i12e21200_app1.png]
